# Production of Stilbenes in Callus Cultures of the Maltese Indigenous Grapevine Variety, Ġellewża

**DOI:** 10.3390/molecules24112112

**Published:** 2019-06-04

**Authors:** Mariella Bonello, Uroš Gašić, Živoslav Tešić, Everaldo Attard

**Affiliations:** 1 Division of Rural Sciences and Food Systems, Institute of Earth Systems, University of Malta, Msida MSD 2080, Malta; mary-anne.bonello@gov.mt (M.B.); everaldo.attard@um.edu.mt (E.A.); 2 University of Belgrade, Faculty of Chemistry, Studentski trg 12-16, P.O. Box 51, 11158 Belgrade, Serbia; urosgasic@chem.bg.ac.rs

**Keywords:** callus, Ġellewża, LC/MS, polyphenols, stilbenes

## Abstract

The production of secondary metabolites in tissue culture has been considered as an alternative to the cultivation and harvesting of crops intended for this purpose. The present study was aimed at the growth of callus and production of polyphenolic compound of callus derived from a Maltese indigenous grapevine variety, Ġellewża. Callus was inoculated onto plant growth regulators-enriched Murashige Skoog media (MSm) to determine whether polyphenols are produced in vitro as well as to determine the best combination of plant growth regulators needed for the production of these metabolites. From results obtained, it was observed that the best callus production was obtained by auxin-enriched MSm. In fact, indole acetic acid and indole acetic acid /6-benzyl aminopurine enhanced biomass accumulation (3.04 g and 3.39 g) as opposed to the others (<1.97 g). On the other hand, parameters showing the presence of flavonoids (tonality, 3.80), particularly anthocyanins (24.09 mg/kg) and total polyphenols (1.42 mg/g), were optimum in the presence of cytokinins, particularly 6-benzyl aminopurine. Analysis for single polyphenols revealed a high amount a particular stilbene: polydatin (glucoside of resveratrol). Resveratrol and other typical polyphenols, found in mature berries, were also found in significant quantities, while the other polyphenolic compounds were found in minimal quantities. This is the first study to describe the production and composition of polyphenols in Ġellewża callus cultures. From the results obtained, it can be seen that this grape tissue is an excellent alternative for the production of polyphenols from the stilbene group, which can be upscaled and exploited commercially.

## 1. Introduction

The Vitaceae family consists of about 14 genera with around 700 species [1], but only the grapevine (*Vitis vinifera*) species are commercially and agriculturally important. *Vitis vinifera* needs hot dry summers to produce its fruit, a climate which is characteristic to the Mediterranean basin. According to Dr John Borg, the Phoenicians introduced the vine and its cultivation to the Maltese Islands. Today, vines are so much acclimatized to the conditions of the islands and terroir, that they even grow wild in valleys and abandoned stretches of rocky land [2].

The National Statistics Office (NSO, Malta)—Agricultural Census carried out in 2010 states that by 2010 there were 614 hectares of permanent vineyards in the Maltese islands, the greater part of which, 428 ha, were in the northern and western part of Malta. There are 74 ha in Gozo, 42 ha in the South Harbour area, 18 ha in the Northern Harbour area and 53 ha in the South Eastern area. There are also a further 121 ha registered as kitchen gardens, 109 ha of which are in Malta, with the remaining 12 ha in Gozo and Comino [3].

*Vitis vinifera* is characterized by a high content of polyphenols acting as natural antioxidants, leading to their investigation by many scientists [4]. The most abundant polyphenolic compounds characteristic for V. *vinifera* are phenolic (hydroxybenzoic and hydroxycinnamic) acids, flavan-3-ols, flavonols, stilbenes, tannins, and anthocyanins [5,6]. Resveratrol, considered to be responsible for the “French paradox” related to wine consumption, is a stilbene derivative that has been most studied for the last decades [7]. Resveratrol has become popular with the discovery of its anticancer potential, reduction in the risk of coronary heart disease and arteriosclerosis, antimicrobial effects, as well as its relation to plant defense [8]. In addition to resveratrol, the family of stilbene derivatives also includes other biologically active compounds such as resveratrol dimers, trimers and tetramers (viniferins), resveratrol glycoside (such as piceid), and others [9].

In a recent study carried out between 2003 and 2005, a total of 58 accessions from around Malta and Gozo were collected and genotyped. Twenty eight different genotypes were identified, eight of which corresponded to imported vine varieties. Of the remaining twenty, the most important were found to be the Ġellewża, Girgentina and Ġennarua [10].

Anthocyanins are natural colorants possessing health benefits and as consumers are becoming aware of the detrimental effects of synthetic colorants in our food, anthocyanin production is becoming one of the most important food industries worth around 200 million dollars annually. The best sources for anthocyanins are plants and the waste grape skins generated by the wine industry. To this end, research with cell cultures has been established for the production of anthocyanins. Moreover, the production of anthocyanins by cell culture has been found to be economically preferable to producing anthocyanins from grape skins. This is because the price of anthocyanin production from grape skins ($2083/kg) is more than double that of cell culture production ($931/kg). This gives further impetus to research given the fact that anthocyanin production from tissue culture is still at its experimental phase and as yet the specific requirements for cell suspension culture complicates matters [11].

The most important local red variety, Ġellewża, is a high producing and very vigorous vine, with pentagonal-orbicular, medium large adult leaves, with three to five lobes. The leaves have a V-shaped petiolar sinus, very sparse or absent hairs on the lower leaf blades, with the leaf sinuses overlapping. Bunches, with one to three wings are long and cylindrical-conical shaped with the berries loosely packed. The berry is small, elliptical with an aromatic taste. The anthocyanin index is 708.24 ± 68.45 mg/kg [12] on average, but there is a high percentage of malvidin which makes for quite stable wines, if not so richly colored. Ġellewża is usually used in, rose, light-red and semi-sparkling wines but is not usually suitable for aged wines [10].

In the past years, the production of phytochemicals by plant cultures in vitro has become very popular [13]. Recent chapter published by Smetanska [14] dealing with optimization of ecological factors for the production of polyphenols by plant in vitro cultures, as well as new developments in the bioprocessing of plant cells. On the other hand, some researchers have focused on compounds which are known to potentially act as antioxidants, and in vitro methods for producing of such compounds [15].

In this study, callus derived from a Maltese indigenous variety, Ġellewża, was inoculated onto plant growth regulator (PGR)-enriched MSm [16] to determine whether polyphenols are produced in vitro as well as to determine the best combination of PGRs needed for the production of these metabolites. Finally, Linear Trap Quadropole-Orbitrap Mass Spectrometry (LTQ-Orbitrap MS) technique was used to study the polyphenolic profile of the expressed phytochemicals.

## 2. Results and Discussion

From the current research work, it was observed that grapevines survive on a media, which is composed of a half-strength MSm supplemented with ascorbic acid, BAP and IAA. This goes in accordance with the study by Roubelakis-Angelakis and Katsirdakis (1990), proving that some grapevine genotypes showed improved growth when placed on half-strength MSm with a high BAP concentration, exceeding 8 µM. The study revealed that there was very little success in callogenesis with leaf explants when incubated on basal media without any PGRs [17].

### 2.1. Biomass Determination

Table 1 illustrates the mass of calluses produced on MSm enriched with different PGRs. Callus cultured on basal medium only did not grow very well. In fact, the second lowest weight of callus was recorded on unenriched media (0.91 g).

Callus grown on media enriched with benzyl aminopurine (BAP) gave the lowest weight of callus recorded (0.41 g, *p* < 0.001 from the rest). There was a significant increase in biomass when BAP was combined with naphthalene acetic acid (NAA) and Indole acetic acid (IAA) (1.21 g and 3.39 g, respectively). IAA-enriched MSm also produced a high biomass (3.04 g). The mass of callus grown on kinetin (KIN) enriched media was relatively low (1.25 g) compared to its combination with IAA (*p* < 0.001), but not statistically different with a KIN/NAA combination (1.09 g). This shows that IAA seems to boost callus biomass given that the highest readings were obtained when IAA was present (3.04 g). This was also evident with BAP, where BAP in combination with IAA produced the highest biomass (3.39 g) whereas on its own BAP produced the lowest biomass. This biomass (about 3.56 g on MSm after 4 weeks) concurs with the one obtained with a grapevine callus (cv. Italia) by Bruno et al., 2006. However, direct comparison cannot be carried out due to differences in culture media and the initial callus weight not being reported in the latter work [18].

### 2.2. Physicochemical Parameters

Table 1 shows the characteristics of the absorbance profile obtained for the different PGR treatments. The color intensity (CI, absorbance units, Au), tonality ratio and anthocyanin (ANTH) content were calculated in triplicate for each treatment.

**Figure 1:**
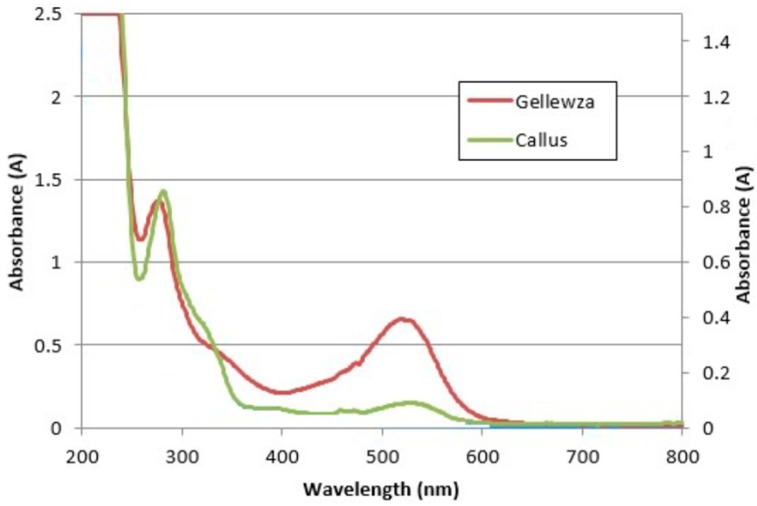
The UV-Vis profile for Ġellewża grapes [12] and Ġellewża callus from the current study.

Color intensity featured distinctively with red grapevine varieties [12]. Color intensity is obtained by the addition of the absorbance values at three wavelengths, i.e., 420, 520, and 620 nm [19]. Values ranged between 0.21 and 3.45 Au, for the whole PGR series. The CI increased above 1.08 Au when PGRs were introduced with the basal medium. However, there was no statistically significant difference between the treatments and the MSm, except for the NAA/BAP combination (*p* > 0.001), which resulted in the highest absorbance value. For this parameter, NAA in combination with a cytokinin exhibited superior CI. Compared to the intact grapes of the Ġellewża variety, callus treated with BAP produced a CI (2.65 Au) that was comparable to the lower end of the CI ranges in the berry (3.06–10.77 Au) [12]. The tonality ratio represents the ratio of the yellow pigments (proanthocyanidins) at 420 nm and the red pigments (anthocyanins) at 520 nm (Figure 1).

In this study, tonality ratios ranged between 1.00 and 3.80. The combination of an auxin with one of the two cytokinins resulted in higher tonality ratios. In the case of IAA, both BAP and KIN enhanced this parameter (3.48 and 3.79 respectively, *p* < 0.05). However, in the case of NAA although the cytokinins enhanced tonality, the resultant difference was not statistically different from the NAA enrichment. It is worth noting that MSm-treated calluses, showed a moderate tonality ratio (2.06). Therefore, it was observed that tonality ratio was lowest on IAA-enriched media but gave the highest result when media was enriched with BAP. Auxin and cytokinin combinations also gave high results. In terms of tonality ratio, the in vitro source (3.80 with BAP and 2.06 with MSm) was superior to the field grape source (0.26–0.39) [12], possibly indicating a high proportion of proanthocyanidins in in vitro callus masses.

Anthocyanins possess health benefits and although water soluble, they are stable colorants. Although anthocyanins are available from a multitude of plants the most widely used source for pigment extraction are the grape skins from wine pressing which otherwise would have been discarded. Grape skins are only available once a year when wine is being made. However, the present study has shown that anthocyanins can be produced from Ġellewża callus, with the greatest amount being produced when the callus was grown on BAP-enriched MSm [16]. It is important to state that the callus was grown in conditions with 16/8 h day/night photoperiod. It should also be stressed that this callus was not treated with any elicitors, so treatment with any elicitor such as methyl jasmonate from *Phaeomoniella chlamydospora* [20,21], or UV [22,23,24] or anything which might stress the vine might very well give very different results. This shows that in vitro anthocyanins could be produced all year round [25].

The anthocyanin content is measured at a wavelength of 520 nm. This is usually an indicator of the intensity of the red pigments in red grapes. The highest anthocyanin content was recorded from callus treated with BAP (24.09 mg/kg). In combination with auxins, BAP exhibited lower anthocyanin accumulations (NAA 22.93 mg/kg; IAA 8.33 mg/kg) indicating that the addition of auxins to BAP hinder anthocyanin production. Callus inoculated on unenriched media resulted in a very low amount of anthocyanins (<1 mg/kg) (*p* < 0.001). Anthocyanin production is linked to the content of sucrose in the medium. At 20 g/L sucrose in medium, moderate anthocyanin production is obtained [26,27]. Callus supplemented with KIN resulted in a low anthocyanin production (11.41 mg/kg) which increased when KIN was combined with IAA and NAA (13.75 mg/kg and 19.78 mg/kg, respectively). This shows that anthocyanin production from a KIN/NAA combination is nearly double that from KIN-enriched media.

A study investigating the anthocyanin content of yogurts supplemented with grape and grape callus extracts from Syrah, Merlot, Chardonnay, Cabernet Sauvignon and callus, found that the highest anthocyanin content was found in callus obtained from Merlot grapes, whilst callus obtained from Chardonnay grapes proved to have the lowest value. This is typical of red grape varieties vis-à-vis white ones [28].

In this present study, anthocyanin content was highest on BAP-enriched media whilst the lowest amounts being recorded on the basal medium. However, the BAP treated callus (24.09 mg/kg) produced inferior anthocyanin content to the field grape source of the same variety (216–976 mg/kg) [12]. This indicates that the red pigments are not highly expressed in tissue culture with this variety.

Polyphenolic content was calculated on a percentage mg/g fresh callus weight basis. In this study callus grown on media-enriched with NAA gave the lowest value (0.43 mg/g FW) followed by that grown on IAA-enriched media (0.57 mg/g FW). Callus grown on unenriched media (0.62 mg/g FW) gave a higher value than that enriched with IAA and NAA. This shows that auxins inhibit polyphenolic production. BAP-enriched media gave the highest reading (1.42 mg/g FW) followed by callus inoculated on media enriched with a combination of BAP and NAA (1.31 mg/g FW) (*p* < 0.001, from the rest). The BAP/IAA combination gave the lowest results (0.97 mg/g FW) where BAP was involved. However, this was significantly higher than other combinations (*p* < 0.05). The combination of KIN with both auxins gave average results (0.96 and 0.95 mg/g FW, respectively), being higher than callus treated with KIN alone (0.74 mg/g FW). It may be concluded that the polyphenolic content was highest on BAP enriched media with the lowest value being recorded from media enriched with NAA. The maximum polyphenolic content for this study (1.42 mg/g FW) is much lower than that reported by Sák et al., 2014 [20], with a grapevine callus (cv. St. Laurent) which amounted to 2.86 mg/g FW in the basal medium cultures. This may be due to varietal differences apart from cultural differences. In fact, for the same Ġellewża variety, the total polyphenolic content of the callus treated with BAP (1.42 mg/g FW) exhibited comparable values to the field grape source (0.754–2.643 mg/g FW). However, the total polyphenolic content in grapevine callus (cv. Öküzgözü) on solid B5 medium with 0.5 g/L of both IAA and BAP (0.58 mg/g FW) [22] was similar to the content in MSm (0.62 mg/g FW) but lower than that in MSm supplemented with IAA/BAP combination (0.97 mg/g FW) for the present study.

A study exploring the fortification of yogurt with various grape and callus extracts found that the highest phenolic content in callus was measured from callus obtained from Merlot whilst callus obtained from Chardonnay was the one with the least phenolic content. The phenolic content of the Merlot callus extract was 15% higher than that of the Chardonnay callus. Tests showed that the free radical capacity of the yogurts was dependent on the age of the yogurt, and decreased over time [28].

### 2.3. Qualitative Liquid Chromatography–Mass Spectrometry (LC/MS) Analysis of Polyphenolics

LC/MS characterization of grape tissue extracts resulted in the detection of 33 polyphenolic compounds in total (Table 2). The identified compounds can be divided into four structurally different groups: (1) phenolic acids and their derivatives (12 compounds); (2) coumarins (2 compounds); (3) stilbenes (14 compounds); and (4) flavonoids (5 compounds). Among all identified compounds, twenty were confirmed using available standards, while the others were identified using high resolution mass spectrometry (HRMS) technique (exact mass search of their deprotonated molecule [M − H]^−^) and MS^2^, MS^3^, and MS^4^ fragmentation behavior, as well as by comparison with the available literature [29,30]. The peak numbers, compound names, retention times (t_R_, min), molecular formulas, calculated and exact masses ([M − H]^−^, *m/z*), mean mass accuracy errors (mDa), as well as major MS^2^, MS^3^, and MS^4^ fragment ions of polyphenolics found in grape tissue extracts are summarized in Table 2. Base peak chromatogram of extract of callus grown on media enriched with benzyl aminopurine (BAP) is shown in Supplementary material (Figure S1).

#### 2.3.1. Phenolic Acid and Their Derivatives

Examination of mass spectra revealed various hydroxybenzoic acid glycosides with characteristic fragmentation by losing of sugar unit (162 Da—hexosyl). Compound **2** at 4.10 min and 299 *m/z* was identified as hydroxybenzoic acid hexoside isomer 1 (Table 2). The MS^2^ base peak of this compound was at 137 *m/z* (deprotonated hydroxybenzoic acid). Further, secondary MS^2^ peaks found at 239, 209, and 179 *m/z* could be formed by specific cleavage of hexose unit (loss of 60, 90, and 120 Da, respectively). Also, a high abundance of the 179 *m/z* suggests that this fragment may be derived from sugar (deprotonated hexose). MS^3^ fragmentation showed only one fragment (MS^3^ base peak at 109 *m/z*), which was obtained by further loss of CO_2_ group (44 Da). Similarly, in the investigated samples, two other compounds were identified, dihydroxybenzoic acid hexoside (compound **1**, 3.03 min) and hydroxybenzoic acid hexoside isomer 2 (compound **4**, 4.71 min).

#### 2.3.2. Coumarins

In our study, two coumarin derivatives (aesculetin and its 6-*O*-glucoside derivative—aesculin) were identified in investigated samples (Table 2). Confirmation of the presence of these two compounds was achieved using available commercial standards. Compound **13**—aesculin (4.94 min and 339 *m/z*) in MS^2^ spectra showed base peak at 177 *m/z* (loss of glucose moiety), which corresponds to deprotonated aesculetin.

#### 2.3.3. Stilbenes

**Figure 2:**
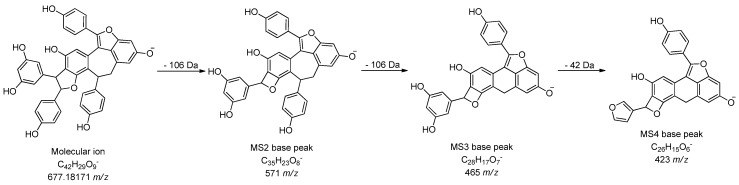
Proposed fragmentation pathway of compound **26**.

As for stilbene derivatives, aside from the two compounds (polydatin and trans-resveratrol) confirmed using available standards, several other derivatives were identified using exact mass search and MS fragmentation. In the investigated samples, 12 stilbene derivatives were detected, including resveratrol dimers, trimer, tetramers, as well as some glycosides. For example, compound **16** with retention time 6.01 min and 551 *m/z* was identified as resveratrol 3,5-*O*-dihexoside. It generated MS^2^ base peak at 389 *m/z*, which was obtained by the loss of one sugar unit (hexosyl residue—162 Da) and MS^2^ secondary peak at 227 *m/z*, corresponding to loss of another sugar unit. MS^3^ base peak at 227 *m/z* and MS^4^ spectral data confirmed the presence of resveratrol as aglycone. Similarly, compounds **19** and **22** were found to have the same accurate masses but slightly different MS^3^ and MS^4^ fragmentation patterns. They were identified as isomers of resveratrol dimer hexoside (Table 2) with same MS^2^ base peak at 453 *m/z*, corresponding to loss of one sugar unit (hexosyl group—162 Da). Resveratrol tetramer (compound **23**) was identified at 8.46 min showing molecular ion at 905 *m/z*, while four derivatives of resveratrol trimer (compounds **21**, **25**, **27**, and **28**) showing different MS data were found at 679 *m/z*. It is interesting to note that compound **26** at 9.39 min and 677 *m/z* was identified as resveratrol trimer with a mass for 2 Da smaller than the other resveratrol trimer isomers, which would mean that the given compound differs in the number of unsaturation. It generated MS^2^ base peak at 571 *m/z*, which was obtained by the loss of 106 Da, and MS^3^ base peak at 465 *m/z* by further loss of 106 Da (C_7_H_6_O). The MS^4^ spectrum showed major fragment ion at 423 *m/z*, corresponding to loss of 42 Da (C_2_H_2_O), which was in accordance with specific fragmentation of stilbene derivatives available in literature [31]. Proposed fragmentation pathway of compound **26** is shown in Figure 2. Similar stilbene derivatives were already identified in the genus *Vitis* [32,33].

#### 2.3.4. Flavonoids

Five compounds from the flavonoid group were identified in the extract of callus grown on different media and presence of all of them was confirmed by comparison with appropriate standards. Among all identified flavonoids, four were aglycones (catechin, myricetin, luteolin, and naringenin) and one was glycoside (quercetin 3-*O*-glucoside).

### 2.4. Quantitative LC/MS Analysis of Polyphenolics

Table 3 shows the individual polyphenolic content (µg/g FW) determined by LC/MS for the different PGR treatments. From the group of flavonoids, it can be concluded that catechin and isoquercetin were most abundant with same concentration (1.04 µg/g FW) and both were quantified in the extract of callus grown on media enriched with IAA. Isoquercetin was not found only in extract of callus grown on media enriched with BAP, while myricetin was detected only in in extract of callus grown on media enriched with KIN. It was observed that Ġellewża callus yielded a good number of metabolites in tissue culture. In general, this experiment proved to be very effective in stilbenes production.

Using the LC/MS technique, several stilbene derivatives (dimers, trimer, tetramers, as well as some glycosides) were identified, semi-quantified and expressed as trans-resveratrol equivalents. The significant metabolite was polydatin (0.34–8.67 µg/g). Polydatin (or piceid) has been found in previous grapevine tissue culture experiments, to be the predominant polyphenolic metabolite [34]. However, in basal medium, there were several undetected metabolites, such as aesculin, syringic acid, myricetin, luteolin and naringenin, when compared to the PGR-treated callus cultures. A study by Sák et al. (2014) [20] showed that vanillic acid and trans-resveratrol were not present in the callus masses, whereas in the present study both metabolites were present in the ranges of 0.01–0.05 µg/g and 0.01–0.70 µg/g respectively. On the other hand, in another study [35], the trans-resveratrol content in Gamborg B-5 basal medium was much higher (0.56 µg/g) compared to that of the present study in MS basal medium (0.16 µg/g).

A number of antioxidants and anti-inflammatory agents have been found, most notably resveratrol, which gave the highest reading when callus was enriched with IAA/BAA followed by the callus enriched with BAA. It is also interesting to note that the high amount of polydatin was produced. Polydatin, the glucoside of resveratrol and its aglucone have shown to modulate interleukin (IL)-6, IL-8 and tumor necrosis factor-alpha gene expression’ and also increased the heat shock protein (Hsp)70B′ gene expression, a Hsp that plays an important role in the cytoprotection and repair of cells and tissues [36].

It is worth noting that when polydatin is used with resveratrol or alone, it increased the release of human β-defensin that plays an important role in the cytoprotection and repair of cells and tissues. This shows that these two metabolites can strengthen the cytoprotective reaction in conditions of stress. This may recommend their use in pharmaceutical or cosmetic preparations [36].

### 2.5. Principal Component Analysis (PCA)

**Figure 3:**
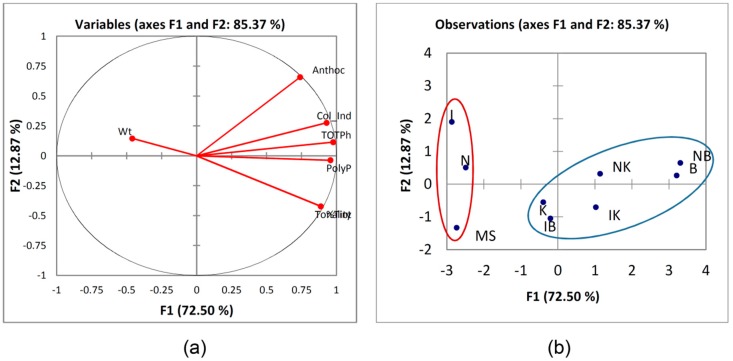
(**a**) The loadings plot and (**b**) The observations plot for the physicochemical parameters studied.

It was observed from the scree plot (data is not shown) that the first component accounted for 72.49% of the total variance. However the parameters studied fall within the principal component, which is in fact the only considerable contributor to the total variance. The loadings plot (Figure 3a) gives the direction of each original variable whilst the scores plot (Figure 3b) shows the position of each location/variety combination. The first factor is loaded heavily on anthocyanin content, color index, total phenolics, polyphenols, tonality and tint which represent a mix of chemical and physical parameters.

The second factor is heavily loaded on weight. It can be seen that all the parameters are within the same factor, except weight which is inversely proportional to tonality and percentage tint and to a certain degree to all other factor 1 parameters. The scores plot (Figure 3b) shows the physicochemical parameters of callus extracts in the space of the two new variables, F1 and F2. Moving along from F1 to the left of the graph it was observed that the callus extracts showed lower physiochemical characteristics from the values shown on the right. The treatments shown on the left-hand side gave lower values than those on the right. Particularly BAP and NAA/BAP showed a distinctive set of characteristics compared to the other single and combination treatments. Callus on MS medium exhibited characteristics that are completely inverse to those of the BAP and NAA/BAP combinations. KIN in combination with auxins exhibited close results to the BAP and NAA/BAP.

## 3. Materials and Methods

### 3.1. Reagents and Standards

Acetonitrile and acetic acid (both of MS grade), methanol (HPLC grade), Folin–Ciocalteu reagent, hydrochloric acid, sodium hypochlorite, and sodium carbonate were purchased from Merck (Darmstadt, Germany). Ultrapure water (Thermofisher Scientific, Bremen, Germany) was used to prepare standard solutions and blanks. Syringe filters (25 mm, nylon membrane, 0.45 μm) were purchased from Supelco (Bellefonte, PA, USA). Analytical standards of phenolic compounds (gallic acid, protocatechuic acid, gentisic acid, *p*-hydroxybenzoic acid, *p*-hydroxyphenylacetic acid, caffeic acid, vanillic acid, syringic acid, *p*-coumaric acid, ferulic acid, aesculin, aesculetin, polydatin, trans-resveratrol, catechin, isoquercetin, myricetin, luteolin, and naringenin) were purchased from Sigma-Aldrich (Steinheim, Germany).

### 3.2. Collection of Samples

Samples were collected from as different locations as possible, particularly in those areas where Ġellewża is reasonably cultivated. Thus, prime agricultural areas were chosen and contacts made with a variety of farmers who could supply or indicate where Ġellewża vineyards were located. The localities of Mġarr (Malta), Mtaħleb (Rabat), Burmarrad and Żabbar were chosen and the relevant farmers were contacted. Samples, two from each locality, were collected when the vines had been pruned. These samples were then placed and wrapped in moist plastic bags and put in dark and cold storage at a temperature of 4 °C.

### 3.3. Forcing

After a period of chilling in the cold room, the scions were taken out, put in moist compost in a plastic bag and placed in the greenhouse at a constant temperature of 24 °C with normal daylight. The cuttings were put in plastic bags to ensure maximum humidity. Following budding and leaf emergence, the cuttings were transferred to pots, watered and closed in plastic bags. The plastic bags were removed when rooting was observed.

### 3.4. Transfer of Cuttings to the Laboratory and Surface Sterilization

When the initial scions exhibited sufficient shoot and root development, the plantlets were ready for tissue culture procedures. The cuttings were clipped off the plants and placed in plastic bags to preserve moisture and then were transferred to the lab. Cuttings were subjected to surface sterilization with 5% hypochlorite solution before being placed on media. The explants were then put on a petri dish. The ends were trimmed off and sections containing buds were cut and put on half-strength basal MSm [16] in the baby jars. It was ensured that the cut end of the explant was in contact with the media. The jars were sealed and kept at a constant temperature of 24 °C, with 16/8 h day/night photoperiod. After about 4 weeks, when the explants showed signs of growth, they were transferred onto fresh media. This was repeated every four (4) weeks. When Ġellewża explants were incubated for some weeks on the MSm, they grew, elongated, and rooted. At times, they produced callus at the base of the stalk. The callus was harvested from the most productive Ġellewża strain and treated with different PGRs.

### 3.5. PGR-Combination Media

In this experiment different combination of PGRs, all with a concentration of 4 mg/L were used (Table 1) with the Murashige Skoog basal medium [16]. For this experiment, callus formed from ĠELM2 was used. Approximately 0.15 g of callus from ĠELM2 accessions were introduced into each jar, for each PGR combination, in triplicates. These were then incubated as previously mentioned and after four weeks the calluses were examined for their polyphenolic content.

### 3.6. Extraction of Polyphenols

From the resultant callus, approximately 0.5 g aliquots from each sample was excised, put in culture tubes and treated with 1 mL of acidified methanol solution (95% methanol + 5% 1M HCl). Samples were then ultrasonicated (Ultrasonicator VWR IP23, Radnor, PA, USA) for 10 min and then centrifuged for 5 min at 1200 rpm (Wisespin CF-10 centrifuge, Wisd Laboratory Instruments, Wertheim, Germany).

### 3.7. Physicochemical Analysis

Samples (100 μL) were diluted in the extractant (1:9) and analysed by UV-Vis spectrophotometry (Lightwave II, WPA) within the range of 200–800 nm, in triplicates. For the determination of the color index, tonality ratio and anthocyanin content (mg/kg), the absorbance readings at 420, 520, and 620 nm were taken [19]. The following calculations were carried out using the formulae below:

(1) $$Colour\;intensity=\left(A_{420}\times DF\right)+\left(A_{520}\times DF\right)+\left(A_{620}\times DF\right)$$

(2) $$Tonality\;Ratio=\;\frac{A_{420}}{A_{520}}$$

(3) $$Anthocyanin\;content\left(\mathrm{mg}/\mathrm{kg}\right)=\frac{1000\times V_{S}\times DF\times A_{520}}{\epsilon }$$

*V_S_* = volume of extracted sample per g of callus sample; *DF* = dilution factor; *ε* = extinction coefficient [58.3 mL(mg.cm)]; *A*_420_, *A*_520_, *A*_620_ = absorbance units (Au) at 420, 520, and 620 nm.

The total polyphenolic content of the calluses was analyzed according to the method described by Attard, 2013 [37]. Briefly, 10 μL of methanolic extracts were transferred to 96-microtitre plate wells, in triplicates and reacted with 100 μL Folin–Ciocalteu reagent (1:10) and 80 μL 1M sodium carbonate. After 20 min incubation at room temperature, the samples were read on a MTP reader (Biotek ELx800, Winooski, VT, USA), at a wavelength of 750 nm. Gallic acid (0‒960 μg/mL) was used as a standard to obtain a calibration curve. The gallic acid equivalents (GAE) were obtained for all samples.

### 3.8. LC/MS Method of Polyphenols Analysis

Separations of polyphenolic compounds were performed using a ThermoFisher Scientific ultra-high performance liquid chromatographic (UHPLC) system consisting of a quaternary Accela 600 pump, Accela Autosampler and analytical Syncronis C18-column (100 × 2.1 mm, 1.7 µm particle size). The mobile phase consisted of (A) water with 0.01% acetic acid in ultrapure water and (B) 100% acetonitrile. The gradient program was as follows: 0.0–1.0 min, 5% B; 1.0–12.0 min, 5–95% B; 12.0–12.2 min, 95–5% B; 12.2–15.0 min, 5% B. The injection volume for all samples was 5 µL and the flow rate was 0.3 mL/min.

This UHPLC system was coupled to a linear ion trap–OrbiTrap hybrid mass spectrometer (LTQ OrbiTrap MS) equipped with heated electrospray ionization probe (HESI-II; Thermo Fisher Scientific, Bremen, Germany). The mass spectrometer operated in negative ion mode and MS spectra were acquired by full range acquisition covering 100–1000 *m/z*. Parameters of the ion source were as in Mudrić et al., 2017 [38]. A data-dependent scan was performed for the fragmentation study by deploying collision-induced dissociation (CID). The ions of interest were isolated in the ion trap with an isolation width of 5 ppm and activated with 35% collision energy levels.

Polyphenolic compounds were identified and quantified in the samples according to the special spectral characteristics: mass spectra, accurate mass, characteristic fragmentation and retention time (*t*_R_). Xcalibur software (version 2.1, Thermo Finnigan LLC, San Jose, CA, USA) was used for instrument control, data acquisition, and data analysis. The molecule editor program, ChemDraw (version 12.0. CambridgeSoft, Cambridge, MA, USA), was used as a reference library to calculate the exact (monoisotopic) masses of compounds of interest. The tentative identification of compounds for which standards were not available was achieved using previously reported MS fragmentation data found in the literature.

Quantification of polyphenolic compounds was carried out by comparing the retention times and accurate mass of compound with available standards. A 1000 mg/L stock solution of polyphenolic standards was prepared in form of methanol solution. This solution was used to make a 10 mg/L solution, which was diluted with methanol to working concentrations of 0.025, 0.050, 0.100, 0.250, 0.500, 0.750 and 1.000 mg/L. In the aim of semi-quantitative comparison (in absence of standards), the amounts of some polyphenolic acid derivatives were expressed as *p*-hydroxybenzoic acid equivalents, while the amounts of stilbene derivatives were expressed as trans-resveratrol equivalents. Calibration curves were formed by plotting the peak areas of the standard solutions against their concentration.

### 3.9. Data Analysis

Color intensity, anthocyanin content, tonality ratio and total polyphenols were investigated with multivariate analysis for all the callus samples. One-way ANOVA with Bonferroni post-hoc test was conducted on all the parameters studied to compare between the treatments applied, using GraphPad Prism ver. 5.0 for Windows (GraphPad Software, San Diego, CA, USA). The parameters were then analyzed with multivariate analysis of all the plant growth regulator-treatment combinations. The correlation matrix was calculated, giving the correlation coefficients between each pair of variables present. To identify variability and to reduce the dimensions of the dataset, principal component analysis (PCA) was performed, using the XLSTAT Version 2011.5.01 software (Addinsoft, New York, NY, USA). Statistical significance was set at *p* < 0.05.

## 4. Conclusions

From this research, it can be concluded that although the lowest biomass weight was recorded on BAP enriched media, it seemed to enhance metabolite production. At subculture time, the callus biomass and metabolite production are inversely related [39]. Although BAP resulted in the lowest biomass accumulation, most of the physicochemical parameters showed to be superior when compared to the other PGR-treated media. Consequently, this shows that tonality ratio, anthocyanin content and total polyphenolics are not dependent on callus weight. It can be seen that there are two phases in callus growth: when callus is physiologically growing and increasing in size and when metabolites are being produced. This process is typical of metabolite-producing calluses [40]. It can be observed that BAP induces metabolite production but slows callus growth. Consequently, callus harvesting for metabolites should be programmed. Auxins tend to enhance the tonality when combined with cytokinins but hinder anthocyanin production when combined with BAP. Cytokinins are important throughout. Auxins on their own hinder metabolite production, but this is not always the case when they are combined with the cytokinins.

As research continues to show that synthetic and artificial food additives are harmful to our health, research is leaning towards the natural production of these additives which are mostly anthocyanins (coloring agents) and other secondary metabolites which have been found to be beneficial to our health.

This study showed a methodology for callus induction in grape tissue of indigenous grapevine variety (Ġellewża) and also production of the bioactive phenolic compounds, of which stilbene derivatives were predominant. This study also showed that both polyphenols and anthocyanins gave the highest reading when callus was inoculated on BAP-enriched media.

It has been shown that plant cell cultures are very good methods for the production of plant chemicals as well as for the production of metabolites that are important to the food, chemical, and pharmaceutical industries [41]. To this end, more research needs to be done to discover ways in which these secondary metabolites can be manufactured. Till now, suspension cell cultures and solid callus cultures seem to be a very expensive way to produce these compounds as research is still in the experimental stage. Further local research, perhaps with the involvement of the relevant interested sectors would enable the utilization and valorization of the indigenous varieties of the grapevine.

## Figures and Tables

**Table 1:** Mean and (±) standard error of the mean for all the treatments for all the callus masses, and physicochemical parameters (color intensity, tonality ratio, anthocyanin content and polyphenolic content).

	Weight	CI	Tonality	ANTH	PolyP
	g	Au	Ratio	mg/kg	mgGAE/g FW
**B**	0.41 ± 0.13	2.65 ± 0.93	3.80 ± 0.40	24.09 ± 9.21	1.42 ± 0.35 *
**I**	3.04 ± 0.78	1.08 ± 0.25	1.00 ± 0.29 **	17.05 ± 6.12	0.57 ± 0.13
**K**	1.25 ± 0.34	1.57 ± 0.19	2.97 ± 0.40	11.41 ± 0.99	0.74 ± 0.06
**N**	1.97 ± 1.19	1.10 ± 0.26	1.70 ± 0.17 **	10.87 ± 2.90	0.43 ± 0.03
**IB**	3.39 ± 0.32	1.23 ± 0.19	3.48 ± 0.51	8.33 ± 1.53	0.97 ± 0.13 *
**IK**	1.97 ± 0.45	2.25 ± 0.64	3.79 ± 0.77	13.75 ± 2.23	0.96 ± 0.12 *
**NB**	1.21 ± 0.48	3.45 ± 0.68	3.74 ± 0.53	22.93 ± 5.79	1.31 ± 0.07 *
**NK**	1.09 ± 0.60	2.06 ± 0.54	3.17 ± 0.42	19.78 ± 7.33	0.95 ± 0.05 *
**MSm**	0.91 ± 0.00	0.00 ± 0.00 **	2.06 ± 0.00	0.00 ± 0.00	0.62 ± 0.00

**B**—Benzyl aminopurine (**BAP**); **I**—indole acetic acid (**IAA**): K—Kinetin (**KIN**); **N**—Naphthalene acetic acid (**NAA**); **IB**—**IAA** & **BAP**; **IK**—**IAA** & **KIN**; **NB**—**NAA** & **BAP**; **NK**—**NAA** & **KIN**; **MSm**—Murashige Skoog medium (i.e., medium without PGRs). All PGRs were added at a concentration of 4 mg/L. * *p* < 0.05; ** *p* < 0.01, from the rest.

**Table 2:** High resolution and negative ion MS^4^ data about identified polyphenolics.

No	Compound Name	*t*_R_, min	Molecular Formula, [M − H]^–^	Calculated Mass, [M − H]^–^	Exact Mass, [M − H]^–^	Δ mDa	MS^2^ Fragments, (% Base Peak)	MS^3^ Fragments, (% Base Peak)	MS^4^ Fragments, (% Base Peak)
**Phenolic acid and their derivatives**									
**1**	**Dihydroxybenzoic acid hexoside**	3.03	C_13_H_15_O_9_^–^	315.07216	315.06992	2.24	**153**^2^(100), 152(50), 109(15), 108(10)	**109**(100)	123(25), 109(10), 85(10), **81**(100)
**2**	**Hydroxybenzoic acid hexoside isomer 1**	4.10	C_13_H_15_O_8_^–^	299.07724	299.07623	1.01	239(70), 209(20), 179(80), **137**(100)	**93**(100)	−
**3**	**Protocatechuic acid ^1^**	4.41	C_7_H_5_O_4_^–^	153.01933	153.01825	1.08	**109**(100), 95(75), 79(20), 59(10)	**81**(100), 68(25), 65(15)	−
**4**	**Hydroxybenzoic acid hexoside isomer 2**	4.71	C_13_H_15_O_8_^–^	299.07724	299.07644	0.80	**137**(100)	**93**(100)	−
**5**	**Gentisic acid ^1^**	5.07	C_7_H_5_O_4_^–^	153.01933	153.01825	1.08	**109**(100), 107(5)	95(10), **81**(100), 65(70)	−
**6**	***p*-Hydroxybenzoic acid ^1^**	5.40	C_7_H_5_O_3_^–^	137.02442	137.02364	0.78	109(10), **93**(100)	**93**(10)	−
**7**	***p*-Hydroxyphenylacetic acid ^1^**	5.65	C_8_H_7_O_3_^–^	151.04007	151.03912	0.95	**136**(100), 95(5)	108(25), **92**(100)	**108**(100)
**8**	**Caffeic acid ^1^**	5.83	C_9_H_7_O_4_^−^	179.03498	179.03366	1.32	**135**(100), 117(10), 91(20), 59(15)	**107**(100), 59(50)	−
**9**	**Vanillic acid ^1^**	5.90	C_8_H_7_O_4_^–^	167.03498	167.03384	1.14	153(10), 152(80), 124(10), **123**(100), 108(20)	**108**(100)	**79**(100)
**10**	**Syringic acid ^1^**	6.03	C_9_H_9_O_5_^−^	197.04555	197.04439	1.16	**182**(100), 153(50), 138(10)	**167**(100), 138(10), 123(5)	**123**(100)
**11**	***p*-Coumaric acid ^1^**	6.75	C_9_H_7_O_3_^–^	163.04007	163.03917	0.90	**119**(100)	119(60), 101(20), 93(25), **91**(100), 72(10)	−
**12**	**Ferulic acid ^1^**	7.12	C_10_H_9_O_4_^–^	193.05063	193.04951	1.12	178(70), **149**(100), 134(50)	**134**(100)	**106**(100)
**Coumarins**									
**13**	**Aesculin (Aesculetin 6-*O*-glucoside) ^1^**	4.94	C_15_H_15_O_9_^–^	339.07216	339.07004	2.12	**177**(100)	177(5), 149(10), **133**(100), 105(10), 89(5)	**89**(100)
**14**	**Aesculetin ^1^**	5.82	C_9_H_5_O_4_^–^	177.01933	177.01828	1.05	147(10), **135**(100), 133(60), 131(40), 105(10)	107(40), **91**(100)	−
**Stilbenes**									
**15**	**Resveratrol hexoside isomer 1**	5.92	C_20_H_21_O_8_^−^	389.12419	389.12232	1.87	**227**(100), 211(15)	**185**(100), 183(40), 159(35), 157(30), 143(20)	−
**16**	**Resveratrol 3,5-*O*-dihexoside**	6.01	C_26_H_31_O_13_^−^	551.17701	551.17462	2.39	**389**(100), 227(15)	**227**(100)	**185**(100), 183(40), 159(35), 157(30), 143(20)
**17**	**Polydatin (Resveratrol 3-*O*-glucoside) ^1^**	6.45	C_20_H_21_O_8_^−^	389.12419	389.12253	1.66	**227**(100)	**185**(100), 183(40), 159(35), 157(30), 143(20)	167(5), 157(10), **143**(100), 117(5)
**18**	**Resveratrol hexoside isomer 2**	7.11	C_20_H_21_O_8_^−^	389.12419	389.12220	1.99	**227**(100)	**185**(100), 183(40), 159(35), 157(30), 143(20)	−
**19**	**Resveratrol dimer hexoside isomer 1**	7.83	C_34_H_31_O_11_^−^	615.18719	615.18396	3.23	**453**(100)	435(20), 411(10), 369(10), **359**(100), 347(40)	341(40), 331(50), **317**(100), 315(80), 291(80)
**20**	***trans*-Resveratrol ^1^**	8.01	C_14_H_11_O_3_^−^	227.07137	227.06966	1.71	**185**(100), 159(30), 143(20)	157(10), **143**(100), 117(5)	**115**(100)
**21**	**Resveratrol trimer isomer 1**	8.21	C_42_H_31_O_9_^−^	679.19736	679.19421	3.15	**585**(100), 491(10)	**491**(100), 479(20), 385(10)	473(40), 447(30), 421(20), 397(25), **385**(100)
**22**	**Resveratrol dimer hexoside isomer 2**	8.39	C_34_H_31_O_11_^−^	615.18719	615.18329	3.90	**453**(100)	**435**(100), 411(70), 409(40), 369(80), 359(30)	435(10), 417(60), 407(40), 393(60), **391**(100)
**23**	**Resveratrol tetramer**	8.46	C_56_H_41_O_12_^−^	905.26035	905.25562	4.73	**811**(100), 717(50), 451(15), 359(15)	**717**(100), 357(10)	699(50), **675**(100), 623(30), 611(60), 357(80)
**24**	**Resveratrol dimer**	8.97	C_28_H_21_O_6_^−^	453.13436	453.13235	2.01	435(10), 369(10), 359(30), **347**(100), 333(40)	329(10), 305(20), **253**(100), 240(30), 225(10)	**225**(100), 209(10)
**25**	**Resveratrol trimer isomer 2**	9.13	C_42_H_31_O_9_^−^	679.19736	679.19434	3.02	661(70), 637(20), **585**(100), 573(90), 451(35)	567(90), 543(50), 491(70), **479**(100), 347(20)	461(70), 435(75), **385**(100), 355(40), 327(30)
**26**	**Resveratrol trimer isomer 3**	9.39	C_42_H_29_O_9_^−^	677.18171	677.17877	2.94	**571**(100), 529(10), 501(20), 465(30), 437(20)	529(30), 501(70), **465**(100), 437(30), 423(20)	447(10), 437(30), **423**(100), 421(60), 371(40)
**27**	**Resveratrol trimer isomer 4**	9.54	C_42_H_31_O_9_^−^	679.19736	679.19391	3.45	661(70), 637(30), **585**(100), 573(80), 451(40)	567(90), 543(40), 491(70), **479**(100), 347(25)	**461**(100), 435(65), 385(90), 355(20), 327(20)
**28**	**Resveratrol trimer isomer 5**	9.86	C_42_H_31_O_9_^−^	679.19736	679.19409	3.27	661(70), 637(15), 585(25), 573(40), **359**(100)	341(50), 331(20), **317**(100), 315(80), 289(70)	299(10), 289(40), **275**(100), 273(70), 261(25)
**Flavonoids**									
**29**	**Catechin ^1^**	5.39	C_15_H_13_O_6_^–^	289.07176	289.06973	2.03	271(5), **245**(100), 205(40), 179(15), 125(5)	227(30), **203**(100), 187(25), 175(10), 161(20)	188(70), 185(20), **175**(100), 161(40), 157(10)
**30**	**Isoquercetin (Quercetin 3-*O*-glucoside) ^**1**^**	6.80	C_21_H_19_O_12_^–^	463.08820	463.08472	3.48	**301**(100), 300(30)	273(25), 257(20), **179**(100), 151(75)	**151**(100)
**31**	**Myricetin ^1^**	8.38	C_15_H_9_O_8_^−^	317.03029	317.02823	2.06	299(10), 273(35), **207**(100), 163(95)	**179**(100), 151(15)	**151**(100)
**32**	**Luteolin ^1^**	8.79	C_15_H_9_O_6_^−^	285.04046	285.03799	2.47	257(40), **241**(100), 217(50), 199(70), 175(70)	255(50), **227**(100), 211(75), 197(35), 183(85)	−
**33**	**Naringenin ^1^**	9.64	C_15_H_11_O_5_^−^	271.06120	271.05905	2.15	177(10), **151**(100)	**107**(100)	**65**(100)

^1^ Conformed using available standards, while the other compounds were identified using HRMS and MS^n^ data available in literature; *t*_R_—retention time; Δ mDa—mean mass accuracy; Peak numbers (No) corresponding to Figure S1. ^2^ MS base peaks that were further fragmented in MS^n^ experiment were marked in bold.

**Table 3:** The individual polyphenolic content (µg/g FW) determined by LC/MS for the different plant growth regulator (PGR) treatments.

No	Compound Name (µg/g FW)	B	I	K	N	IB	IK	NB	NK	MSm
**Phenolic acid and their derivatives**										
**1**	**Dihydroxybenzoic acid hexoside ^2^**	0.37	0.06	0.08	0.01	1.00	0.34	0.52	0.30	0.05
**2**	**Hydroxybenzoic acid hexoside isomer 1 ^2^**	1.89	0.32	0.19	0.36	0.32	0.21	2.27	1.66	0.07
**3**	**Protocatechuic acid ^1^**	0.25	0.19	0.85	0.09	0.13	0.20	0.19	0.27	0.75
**4**	**Hydroxybenzoic acid hexoside isomer 2 ^2^**	–	–	0.01	–	0.17	0.13	0.48	0.57	–
**5**	**Gentisic acid ^1^**	0.56	0.96	0.22	0.25	1.03	1.01	0.67	0.51	0.75
**6**	***p*-Hydroxybenzoic acid ^1^**	0.32	0.12	0.17	0.11	0.18	0.31	0.52	0.36	0.07
**7**	***p*-Hydroxyphenylacetic acid ^1^**	0.01	0.02	0.01	–	0.01	–	0.01	–	0.01
**8**	**Caffeic acid ^1^**	0.06	0.09	0.11	0.08	0.06	0.11	0.13	0.09	0.07
**9**	**Vanillic acid ^1^**	0.01	0.05	0.03	0.05	0.02	0.02	0.01	0.02	0.01
**10**	**Syringic acid ^1^**	0.08	0.08	0.09	0.08	–	0.08	0.09	0.08	–
**11**	***p*-Coumaric acid ^1^**	0.06	0.04	0.08	0.06	0.10	0.15	0.07	0.07	0.03
**12**	**Ferulic acid ^1^**	0.02	0.02	0.01	0.03	0.02	0.02	0.03	0.02	0.01
**Coumarins**										
**13**	**Aesculin (Aesculetin 6-*O*-glucoside) ^1^**	0.28	–	0.30	0.01	0.03	0.06	0.04	0.03	–
**14**	**Aesculetin ^1^**	0.01	0.01	0.02	0.01	–	–	–	–	0.01
**Stilbenes**										
**15**	**Resveratrol hexoside isomer 1 ^3^**	–	–	0.01	–	0.01	–	0.02	–	0.01
**16**	**Resveratrol 3,5-*O*-dihexoside ^3^**	0.20	0.08	0.30	0.28	0.26	0.40	0.25	0.41	0.30
**17**	**Polydatin (Resveratrol 3-*O*-glucoside) ^1^**	0.34	4.10	2.49	1.14	8.67	1.93	4.27	1.58	3.43
**18**	**Resveratrol hexoside isomer 2 ^3^**	0.38	1.10	0.63	0.46	0.71	0.53	0.47	0.25	0.65
**19**	**Resveratrol dimer hexoside isomer 1 ^3^**	0.19	0.06	0.24	0.02	0.29	0.11	0.88	0.05	0.05
**20**	***trans*-Resveratrol ^1^**	0.55	0.05	0.39	0.01	0.70	0.45	0.37	0.24	0.16
**21**	**Resveratrol trimer isomer 1 ^3^**	0.33	–	0.23	–	0.38	0.91	0.08	0.03	0.01
**22**	**Resveratrol dimer hexoside isomer 2 ^3^**	0.20	0.05	0.19	0.02	1.02	0.46	0.23	0.08	0.05
**23**	**Resveratrol tetramer ^3^**	0.52	–	0.19	–	0.14	0.22	0.82	0.27	–
**24**	**Resveratrol dimer ^3^**	2.30	–	1.58	0.02	1.08	2.28	1.32	0.18	0.03
**25**	**Resveratrol trimer isomer 2 ^3^**	3.32	0.01	1.69	0.01	0.71	2.73	2.55	0.10	–
**26**	**Resveratrol trimer isomer 3 ^3^**	7.23	0.05	3.85	0.01	2.31	5.33	2.60	0.32	0.04
**27**	**Resveratrol trimer isomer 4 ^3^**	1.10	–	0.30	–	0.28	0.43	0.90	0.08	0.01
**28**	**Resveratrol trimer isomer 5 ^3^**	0.80	–	0.11	–	0.06	0.93	0.36	0.36	0.01
**Flavonoids**										
**29**	**Catechin ^1^**	0.15	1.04	0.11	0.14	0.78	0.10	0.41	0.12	0.09
**30**	**Isoquercetin (Quercetin 3-*O*-glucoside) ^1^**	–	1.04	0.12	0.11	0.18	0.10	0.13	0.08	0.26
**31**	**Myricetin ^1^**	–	–	0.17	–	–	–	–	–	–
**32**	**Luteolin ^1^**	0.04	0.10	0.04	0.02	0.03	0.03	0.04	0.03	–
**33**	**Naringenin ^1^**	0.01	0.08	0.01	–	0.01	0.04	0.04	0.02	–

^1^ Quantified using available standards. Amount of phenolic acid derivatives; ^2^ were expressed as *p*-hydroxybenzoic acid equivalents, while the amount of stilbene derivatives; ^3^ were expressed as *trans*-resveratrol equivalents; “−” not detected. Peak numbers corresponding to Figure S1.

## Supplementary Materials

The following are available online at https://www.mdpi.com/1420-3049/24/11/2112/s1, Figure S1: Base peak chromatogram of callus enriched with BAP.
