# Supplementary material for: Production of Stilbenes in Callus Cultures of the Maltese Indigenous Grapevine Variety, Ġellewża
Source: Molecules. 2019 Jun 4;24(11):2112. doi: 10.3390/molecules24112112 (PMC6600261; doi:10.3390/molecules24112112)
Supplement: Supplementary file 1 [file molecules-24-02112-s001.pdf]

Article

Production of Stilbenes in Callus Cultures of the Maltese Indigenous Grapevine Variety, Ġellewża

Mariella Bonello [1], Uroš Gašić [2], Živoslav Tešić [2,*] and Everaldo Attard [1]

[1] Division of Rural Sciences and Food Systems, Institute of Earth Systems, University of Malta, Msida MSD 2080, Malta; mary-anne.bonello@gov.mt (M.B.); everaldo.attard@um.edu.mt (E.A.)

[2] University of Belgrade, Faculty of Chemistry, Studentski trg 12-16, P.O. Box 51, 11158 Belgrade, Serbia; urosgasic@chem.bg.ac.rs

* Correspondence: ztesic@chem.bg.ac.rs; Tel.: +381113336733

Received: 14 May 2019; Accepted: 29 May 2019; Published: 4 June 2019

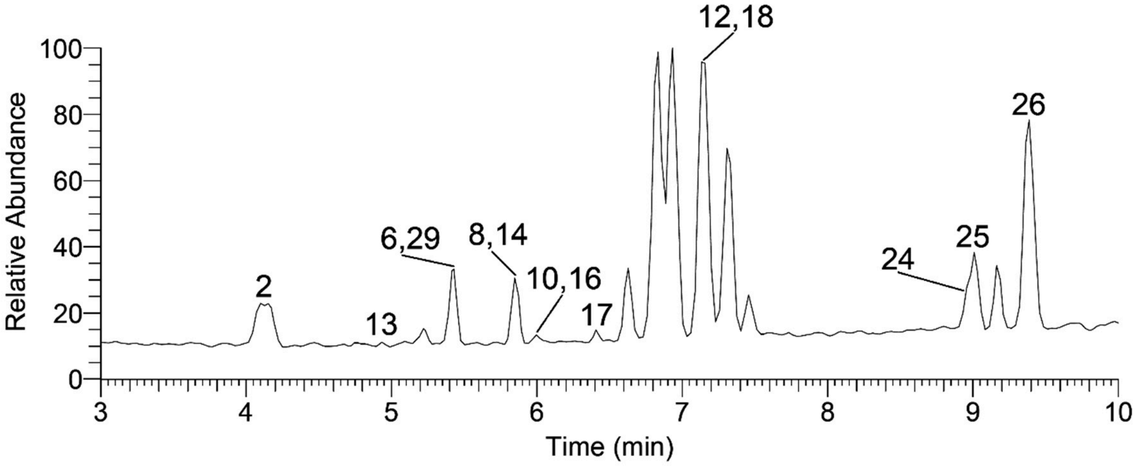

Figure S1. Base peak chromatogram of callus enriched with~BAP.
